# Reducing Water Availability Impacts the Development of the Arbuscular Mycorrhizal Fungus *Rhizophagus irregularis* MUCL 41833 and Its Ability to Take Up and Transport Phosphorus Under *in Vitro* Conditions

**DOI:** 10.3389/fmicb.2018.01254

**Published:** 2018-06-11

**Authors:** Olivia Le Pioufle, Stéphane Declerck

**Affiliations:** Earth and Life Institute, Applied Microbiology, Mycology, Université catholique de Louvain, Louvain-la-Neuve, Belgium

**Keywords:** arbuscular mycorrhizal fungi, polyethylene glycol (PEG), drought stress, hyphal development, phosphorus uptake, phosphorus transport

## Abstract

Climate change scenarios predict a higher variability in rainfall and an increased risk of water deficits during summers for the coming decades. For this reason, arbuscular mycorrhizal fungi (AMF) and their mitigating effects on drought stress in plants are increasingly considered in crop management. However, the impact of a decrease in water availability on the development of AMF and their ability to take up and transport inorganic phosphorus (Pi) to their hosts remain poorly explored. Here, *Medicago truncatula* plantlets were grown in association with *Rhizophagus irregularis* MUCL 41833 in bi-compartmented Petri plates. The system consisted in associating the plant and AMF in a root compartment (RC), allowing only the hyphae to extend in a root-free hyphal compartment (HC). Water availability in the HC was then lowered by increasing the concentration of polyethylene glycol-8000 (PEG-8000) from 0 to 10, 25, and 50 g L^-1^ (corresponding to a slight decrease in water potential of -0.024, -0.025, -0.030, and -0.056 Mpa, respectively). Hyphal growth, spore production and germination were severely impaired at the lowest water availability. The dynamics of Pi uptake by the AMF was also impacted, although total Pi uptake evaluated after 24 h stayed unchanged. The percentage of metabolically active extraradical hyphae remained above 70%. Finally, at the lowest water availability, a higher P concentration was observed in the shoots of *M. truncatula*. At reduced water availability, the extraradical mycelium (ERM) development was impacted, potentially limiting its capacity to explore a higher volume of soil. Pi uptake was slowed down but not prevented. The sensitivity of *R. irregularis* MUCL 41833 to a, even small, decrease in water availability contrasted with several studies reporting tolerance of AMF to drought. This suggests a species or strain-dependent effect and support the necessity to compare the impact of water availability on morpho-anatomy, nutrient uptake and transport capacities of other, potentially more drought-tolerant (e.g., isolated from dry environments) AMF.

## Introduction

Drought associated to extreme heat has been responsible for a significant drop in yield of crops and cultivable surfaces over the last 40 years (Lesk et al., 2016). Projected climate changes indicate a higher variability in rainfall in the coming decades, with an increasing risk of water deficits during summers, while water resources for irrigation will be at best maintained. Enhancing crops resilience to drought is thus a priority to secure nowadays and future’s food production (Wheeler and von Braun, 2013).

There are various soils, water and crop management practices which, together with improved genotypes, have the potential to increase water use efficiency of crops. For instance, irrigation is a practical solution to increase crop yield, although an intensification of this method is not viable on the long term as freshwater resources are limited (Elliott et al., 2014). Irrigation methods that avoid salinization of soil, soil leaching, and improve water use efficiency are alternatives, but are not always affordable for smallholders (Wezel et al., 2014; Xie et al., 2014). Soil microorganisms living in close relations with plant roots might also play a role in plant water stress mitigation via various mechanisms (see for review Kim et al., 2012). Among these below-ground inhabitants are the beneficial and naturally occurring arbuscular mycorrhizal fungi (AMF) (Cardoso and Kuyper, 2006; Rodriguez and Sanders, 2014).

Arbuscular mycorrhizal fungi are obligate root symbionts that have co-evolved with plants for an estimate of 400 million years (Parniske, 2008). They are nowadays associated with an approximate of 72% of Angiosperms (Brundrett, 2009) in almost all ecosystems (Smith and Read, 2008). Their beneficial effects on plant nutrition and growth as well as on biotic and abiotic stresses alleviation have been amply described (Miransari, 2014; Abdel Hamed Abdel Latef et al., 2016; Lenoir et al., 2016; Plouznikoff et al., 2016) so that they are today considered as essential components of agro-ecosystems. In the recent years, many studies have focused on the role of AMF in plant water stress mitigation (Aroca and Ruiz-Lozano, 2009; Jayne and Quigley, 2014; Fernández-Lizarazo and Moreno-Fonseca, 2016; Farooq et al., 2017). Under dry environments, AMF have been reported to regulate plant physiology and metabolism (Wu and Zou, 2017) by changing absissic acid/cytokinin hormone balance (Goicoechea et al., 1997) and by protecting the host plants from oxidative damages under osmotic stress conditions (Porcel and Ruiz-Lozano, 2004). They have been shown to improve osmotic adjustment (Wu and Xia, 2006) and water lift from roots to shoots via higher water apoplastic flow (Bárzana et al., 2012). They have also been reported to increase cell to cell water transport through increased expression of aquaporin membrane proteins (Aroca et al., 2007; Ruiz-Lozano et al., 2009). Increased drought tolerance of AMF-colonized plants has also been associated to an improved nutrition, especially inorganic phosphorus (Pi) that is transported by the extraradical hyphae of the fungus to the root cells (Nelsen and Safir, 1982; Bethlenfalvay et al., 1988; Fitter and Nichols, 1988). The extraradical hyphae can increase the access of plants to soil water (Sánchez-Díaz and Honrubia, 1994; Ahmad Khan et al., 2003) and increase soil water retention through glomalin excretion (Augé, 2001; Wu et al., 2008).

Despite the quite large number of studies reporting the effects of AMF on plant physiology and growth under drought stress conditions, little is known on the impact of decreased water availability on the development of the fungus and its capacity to take up and transport Pi to plants, an essential attribute of these fungi. Any change with respect to their growth and development or capacity to provide the host plant with nutrients would indeed partly compromise their agro-ecological value. Today, most studies have been conducted in pots and a few in bi-compartmented systems making it possible to differentiate Pi uptake by the plant from that of the fungus (Thingstrup et al., 2000; Jansa et al., 2003; Merrild et al., 2013). However, these systems do not allow the non-destructive observation of AMF development. Moreover, the dynamics of Pi uptake by the sole extraradical mycelium (ERM) of the fungus is nearly impossible to assess due, among others, to the presence of unwanted microbial contaminants.

*In vitro* cultivation systems associating excised roots (Declerck et al., 2005) or whole plants (Voets et al., 2005) in mono or bi-compartmented Petri plates (St-Arnaud et al., 1996) are more indicated to overcome some of these issues. They have been used to study the development (Bago et al., 1996, 1998; St-Arnaud et al., 1996; Declerck et al., 2005) and architecture (Bago et al., 1998; de la Providencia et al., 2005; Voets et al., 2006) of the ERM as well as the intimate plant-AMF association under abiotic stress conditions (e.g., heavy metals – Pawlowska and Charvat, 2004; potentially toxic elements such as chromium – Gil-Cardeza et al., 2017; salt – Jahromi et al., 2008; Aroca and Ruiz-Lozano, 2009; fungicides – Zocco et al., 2011; polyaromatic hydrocarbons (PAHs) – Calonne et al., 2012). In particular, the utilization of bi-compartmented Petri plates (with the fungus developing in a root-free compartment) has allowed to monitor Pi uptake and transport in presence of fungicides (Zocco et al., 2011) or PAHs (Calonne et al., 2014).

A few *in vitro* studies have also been focused on the effects of water stress on the AMF. Indeed, mannitol has been reported to decrease germination and germ tube growth of *Funneliformis mosseae* (Estaun, 1989) on gelled medium. Glycerol has also been reported to decrease mycelium biomass, spore production and spore size of *R. irregularis* DAOM 197198 (Hammer and Rillig, 2011). More recently, osmotic stress induced by the application of polyethylene glycol (PEG-8000) in liquid Minimum (M) medium upregulated the expression of the fungal aquaporins GintAQP1 and GintAQP2 of *Rhizophagus irregularis* DAOM 197198 associated to transformed *Daucus carota* roots in bi-compartmented Petri plates (Li et al., 2013). PEG is an inert and low toxic polymer (Lawlor, 1970; Mexal and Reid, 1973), thus representing an adequate option to create water shortage in gelled medium. Over the last decades, it has become the principal compound used to study physiological response of plants cultivated *in vitro* to water stress (Rai et al., 2011). Therefore, it appears to be a good candidate to investigate the role of water deficit on the development and architecture of AMF and on the uptake and transport of Pi.

In the present work, we first conducted a preliminary study to assess the water potential achieved by increasing concentrations of PEG in liquid medium. We then evaluated and compared the effects of PEG-induced decreased water availability on the development (i.e., spore germination and ERM development) and on the Pi uptake and transport of *R. irregularis* MUCL 41833 associated to *Medicago truncatula*. Albeit water potential achieved under our experimental conditions are much higher than encountered under dry environments, we hypothesize that slight changes in water availability can trigger differential response of the fungus.

## Materials and Methods

### Biological Material

Seeds of *Medicago truncatula* Gaertn. cv. Jemalong A 17 (SARDI, Australia) were surface-disinfected by immersion in sodium hypochlorite (8% active chloride) for 10 min and rinsed in sterilized (121°C for 15 min) deionized water. Seeds were germinated in Petri plates (90 mm diameter) containing 35 ml of modified Strullu–Romand (MSR) medium (Declerck et al., 1998) without vitamins and supplemented with 10 g L^-1^ sucrose and 3 g L^-1^ Gelrite (Merck & Co., Kenilworth, NJ, United States). The MSR medium was adjusted to pH 5.5 before sterilization (121°C for 15 min). The Petri plates were incubated at 27°C in the dark. The seedlings were ready to use 4 days following seed germination.

The AMF *R. irregularis* (Błaszk., Wubet., Renker and Buscot) C. Walker and A. Schüßler comb. nov. MUCL 41833 was supplied by the Glomeromycota *in vitro* collection (GINCO^1^). The fungus was grown in bi-compartmented Petri plates (90 mm diameter) in association with *M. truncatula* plantlets following the method developed by Voets et al. (2009).

### Preliminary Experiment: Water Potential of MSR Medium Supplemented With Increasing Concentrations of Polyethylene Glycol

Polyethylene glycol 8000 (PEG-8000; Applichem, Darmstadt, Germany) was used to induce a decrease in water availability. The efficiency of PEG in decreasing the water potential and thus water availability of the MSR medium was determined as follows: liquid MSR medium without PEG or supplemented with 10, 25, or 50 g L^-1^ PEG was prepared. The dew point at each concentration was determined using a vapor pressure osmometer (VAPRO^®^ 5520, Wescor Inc., Logan, UT, United States). The osmolarities obtained from the different solutions were then converted into water potentials (ψ). Response curve of water potential to PEG concentrations was finally built using a quadratic model in the JMP statistic software (JMP^®^Pro 12.1.0) from SAS. Quality of fit between the model and the data was evaluated by computation of the *R*^2^.

For the various assays described below, PEG at the various concentrations was mixed with sterilized (121°C for 15 min) liquid or solid (with 3% of Gelrite) MSR medium without sucrose and vitamins. Solubilization of PEG in the MSR medium was done with a sterilized magnetic rod and at a temperature of 45°C to prevent medium solidification before pouring in the Petri plates.

### Preparation of the Mycelium Donor Plants to Study the Hyphal Development, Spore Production, and Pi Uptake by the AMF

Spores (±100) of *R. irregularis* MUCL 41833 were associated to a 4-day-old *M. truncatula* plantlet in the root compartment (RC) of a 90 mm diameter bi-compartmented Petri plate [named Mycelium Donor Plant (MDP) *in vitro* culture system – see for details Voets et al., 2009]. The roots were plated on the MSR medium with the shoot extending outside the MDP *in vitro* culture system via a hole cautiously plastered with silicon grease (VWR, Radnor, PA, United States) to avoid contaminations. The RC and adjacent hyphal compartment (HC) were filled with 25 ml MSR medium without sucrose and vitamins and solidified with 3 g L^-1^ gelrite. The MDP *in vitro* culture systems were then transferred to a growth chamber at 22/18°C (day/night), 70% relative humidity (RH), a photoperiod of 16 h day^-1^ and a photosynthetic photon flux (PPF) of 225 μmol m^-2^ s^-1^. The systems were covered with dark plastic sheets to protect *M. truncatula* roots and AMF from the light, while the shoots developed under light conditions. To nourish the plantlet, RC was supplied every week with approximately 10 ml of fresh MSR medium using a 25 ml sterile pipette (VWR). To allow the passage of the pipette, a hole in the lid above the RC was made using a hot cork borer and was closed after pipetting using a double layer of sterilized plaster (Micropore^TM^, 3M^TM^, Maplewood, MN, United States). Hyphae started to cross the partition wall separating the RC from the HC within 8 to 12 weeks. Two 4-day-old *M. truncatula* plantlets were subsequently transferred in the HC of each system to allow pre-mycorrhization of new plantlets. Roots were plated on the MSR medium without sucrose and vitamins and shoots extended outside the system via two holes, similarly plastered with silicon grease to avoid contaminations. The new plantlets were homogenously colonized within 12 days.

### Impact of Decreasing Water Availability on Hyphal Development and Spore Production of *R. irregularis* MUCL 41833

Single 12-day-old pre-mycorrhized *M. truncatula* plantlets were plated in the RC of new bi-compartmented Petri plates using the same technique as for the preparation of MDP systems (see above). Using a sterile pipette, the RC was supplied with 15 ml of solid MSR medium without sucrose and vitamins every 2 weeks during the whole experiment. After 9 weeks, 24 systems showing profuse ERM development in the HC were selected. To allow homogenous growth of the AMF in this compartment, the MSR medium was cut 2 mm away from the partition wall and removed. The HC was then supplemented with 25 ml MSR medium lacking sucrose and vitamins and solidified with 3 g L^-1^ of gelrite in absence (Control^-PEG^ treatment) or supplemented with 10, 25, and 50 g L^-1^ PEG (PEG^10^, PEG^25^, and PEG^50^ treatments, respectively). Hyphae re-colonized the MSR medium within 4 weeks. Hyphae and spore densities were homogenous along the partition wall, although their respective length and number gradually decreased along the growing front. Hyphal length and spores number were counted under binocular microscope (40× magnification) in three 10 mm^2^ squares located in the middle of the HC, perpendicular to the partition wall and parallel to the growing axis. Hyphal length (L) was estimated using the Newmann’s formula modified by Marsh (1971):

$$\begin{array}{c} L\;(\mathrm{cm})=N\times (11/14)\times \mathrm{grid}\;\mathrm{unit}\;(\mathrm{cm}) \end{array}$$

where N is the number of hyphal intersections with the grid, 11/14 is the ideal size of the grid sides that gives direct estimation of length in cm and grid unit = 1 cm.

Spores number was summed over the three squares (Declerck et al., 2003). Six replicates were considered per treatment.

### Impact of Decreasing Water Availability on Spore Germination and Germ Tube Length of *R. irregularis* MUCL 41833

Spores of a two-month-old MDP *in vitro* culture of *R. irregularis* MUCL 41833 were isolated from the MSR medium following solubilization of the gelled medium with 10 mM filter-sterilized sodium citrate buffer (Doner and Becard, 1991). Spores were separated with forceps and placed single with a micropipette in each well of two 24-well tissue culture plates (VWR, Radnor, PA, United States) containing 2 ml of solid MSR medium without sucrose and vitamins and without PEG (Control^-PEG^ treatment) or supplemented with 10, 25, and 50 g L^-1^ PEG (PEG^10^, PEG^25^, and PEG^50^ treatments, respectively). The 24-well tissue culture plates were then sealed with cellophane (Toppits^®^, Melitta Group, Germany) and incubated in the dark at 27°C. Spores germination was estimated at regular intervals for 110 days and hyphal length of germinated spores was similarly evaluated at day 110 under binocular microscope (30× magnification).

For Control^-PEG^, PEG^10^ and PEG^25^ but not PEG^50^ treatment, Gompertz model was fitted to the germination counts by non-linear (NLIN) regression using Gauss–Newton minimization algorithm in SAS software. Effect of the treatment was evaluated by testing a significant reduction of the residual sum of square when parameterizing with and without an effect of the treatment using the Fisher–Snedecor *F*-test with 1 and *t*-4 df (Seber and Wild, 1989; Declerck et al., 2001): $Fr=\frac{({SSEr-SSE}_{f})/q}{{\mathrm{MSE}}_{f}}$ where SSE_r_ is the residual sum of square in reduced model and SSE_f_ in the full model, MSE_f_ = residual mean square in the full model.

Parameter estimates of the model in the different treatments were further compared to assess an effect on the dynamics of germination in the HC. Confidence intervals were built around the following estimated parameters: coefficient “a” referred to the cumulated number of germinated spores when the plateau phase was reached, “b” referred to the maximum spore germination rate (tangent of the curve at the inflection point), and “c” referred to the lag time (time to the intersection between the tangent at the inflection point and the time axis).

### Impact of Decreasing Water Availability on Pi Uptake by *R. irregularis* MUCL 41833, SDH Activity of Hyphae, Plant Biomass, and Phosphorus Concentration

**FIGURE 1:**
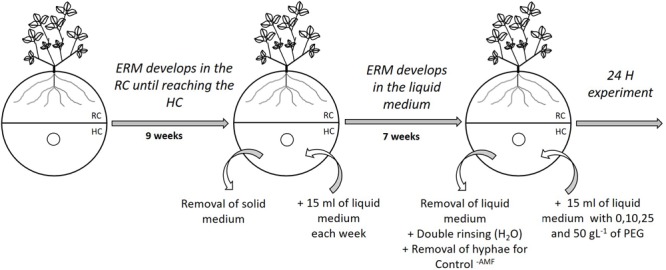
Schematic representation of the preparation of bi-compartmented Petri plates to assess the impact of decreasing water availability on the inorganic phosphorus uptake by *Rhizophagus irregularis* MUCL 41833. The fungus is associated with *Medicago truncatula* roots in the root compartment (RC) and extraradical mycelium (ERM) extends in the hyphal compartment (HC). Small circles represent the opening made in the lid of the Petri plate used for adding modified Strullu–Romand (MSR) medium. Addition of solid MSR medium in the RC is not represented in the scheme.

Phosphorus uptake by *R. irregularis* MUCL 41833 was measured indirectly by monitoring Pi concentration remaining in the medium of the HC of the MDP *in vitro* culture systems. This approach cannot exclude that some of the Pi is adsorbed on the hyphae, although most studies conducted *in vitro* (e.g., Nielsen et al., 2002; Zocco et al., 2011; Calonne et al., 2014; Gil-Cardeza et al., 2017) clearly show that the vast majority of Pi is taken up and transported by the mycelium. Single 12-day-old pre-mycorrhized *M. truncatula* plantlets were plated in the RC of bi-compartmented Petri plates using the same technique as for the preparation of MDP systems (see above). The RC was supplied with 15 ml of solid MSR medium without sucrose and vitamins every 2 weeks during the whole experiment. A profuse ERM was produced in the RC within 9 weeks and started to cross the partition wall separating the RC from the HC (**Figure 1**). At that time, the gelled MSR medium in the HC was entirely removed and replaced by 15 ml of liquid MSR medium without sucrose and vitamins (**Figure 1**). A hole in the lid of the system was made above the HC, similarly to the procedure described in Section “Preparation of the Mycelium Donor Plants to Study the Hyphal Development, Spore Production, and Pi Uptake by the AMF.” Using a 25 ml sterile pipette, the HC was then supplied weekly with approximately 15 ml of liquid MSR medium. Profuse hyphal regrowth in the HC was obtained within 7 weeks. The MDP *in vitro* culture systems were then separated in four homogenous groups based on the surface covered by the hyphae in the HC. Hyphal surface was estimated following the method described by Voets et al. (2009) by tracing on a transparent sheet the growing front constituted by the hyphal network. This measure confirmed no significant differences (*P* = 0.6395) between treatments.

Four treatments, each with six replicates, were considered: MDP *in vitro* culture systems without PEG in the HC (Control^-PEG^ treatment) or supplemented with 10, 25, and 50 mg L^-1^ PEG (PEG^10^, PEG^25^, PEG^50^ treatments, respectively). A control (six replicates) without mycelium in the HC (Control^-AMF^ treatment) was also considered by cutting and removing the ERM from the HC. Liquid MSR medium was retrieved from the HC and the compartment was rinsed two times with sterilized deionized water (**Figure 1**). Fifteen ml of fresh liquid MSR medium was added to the Control^-AMF^ and Control^-PEG^ treatments, whereas liquid MSR medium containing 10, 25, and 50 g L^-1^ PEG was added to the other treatments (**Figure 1**). The Pi concentration in the HC at the start of experiment was determined by inductively coupled plasma atomic emission spectroscopy (ICP-AES; ICP 6500, Thermo-Scientific). The value was 0.59 mg L^-1^ and total P content 0.09 mg.

For Pi concentrations analysis in the HC, 0.5 ml of MSR medium was sampled every 2 h during 24 h from the HC of each system. The samples were diluted (1/10) and digested with 20 μl of nitric acid (65%, Suprapur^®^, Merck, Germany) and finally analyzed by ICP-AES. Data of Pi concentration obtained in ppm were subsequently converted into mg L^-1^ and standardized as in Garcés-Ruiz et al. (2017). Data were finally transformed into % of Pi remaining in the medium which is the percentage of the Pi concentration remaining in the medium of the HC at time 2, 4, 6, 8, 12, 18, 24 h relative to the Pi concentration in the medium of the HC at time T_0_:

$$\begin{array}{c} \%\mathrm{of}\;Pix=([Pix]t+[Pic]t)/[Pix]t0\times 100 \end{array}$$

where [*Pix*] = Pi concentration in the medium of the HC for the sample *x* considered

*t* = time considered (2, 4, 6, 8, 12, 18, or 24 h after the start of the experiment)

*t*0 = 0 h before the start of the experiment

[*Pic*] = Mean Pi concentration measured over six replicates in the Control^-AMF^ treatment

At the end of the sampling phase, the ERM in the HC was harvested from the liquid MSR medium and stained for succinate dehydrogenase (SDH) detection. The SDH staining method enabled to evaluate the proportion of metabolically active hyphae (Saito, 1995; Vierheilig et al., 2005). Hyphae were harvested and incubated overnight at room temperature in the dark in a freshly prepared staining solution containing Tris–HCl (50 mM), MgCl_2_ (0.5 mM), sodium succinate (25.1 mM) and Nitroblue tetrazolium (1.22 mM). The samples were then washed with deionized water, counterstained in 0.1% acid fuchsin in lactic acid for 15 min and transferred to lactoglycerol for de-staining. Hyphae samples were mounted on microscope slides with lactoglycerol. The precipitation of substrate after enzymatic reaction was assessed under a bright field dissecting microscope (Olympus SZ, Olympus Optical, Germany) at 500× magnification. For each sample, an approximate of 100 hyphal intersections was observed. According to Van Aarle et al. (2002), intersections of hyphae were classified as active or non-active following substrate precipitation or not.

Following harvest, shoots and roots were separated and dry weights evaluated after 48 h at 45°C in an oven. The dried shoots and roots material were then grinded and subsamples heated overnight at a temperature of 60°C with 4 ml of nitric acid 65% followed by an evaporation step at 120°C. Subsamples were then dissolved in 2 ml solution of aqua regia (3/4 HCL 38% for 1/4 HNO_3_ 65%). The solutions were filtered and diluted in 25 ml of purified MilliQ (Millipore^TM^) water for P concentration analysis via ICP-AES.

### Statistical Analysis

Data analysis was performed with JMP or SAS statistical software (SAS Institute Inc., Cary, NC, United States) with a 5% α-threshold. Pairwise comparisons following chi-square tests were analyzed using Bonferroni’s correction. Significant differences between treatments for water potential measurements, hyphal lengths, shoots and roots dry biomass, shoots and roots P concentrations and content as well as for hyphal surfaces, and relative water contents were tested using analysis of variance with one factor (ANOVA1) followed by a *post hoc* Tuckey (α = 5%) test. Levene’s test was run prior to the ANOVA analysis to confirm homogeneity of variance in the distribution. Number of spores produced were tested using a likelihood ratio test for negative binomial model. Data corresponding to the % of Pi remaining in the medium were submitted to an arcsin^-1^ transformation and submitted to repeated-measures ANOVA with REML estimation where “Treatment” was regarded as a fixed factor and “Time” as random factor. Additionally, % of Pi remaining in the medium was compared for each time using ANOVA1 test as described above. Finally, SDH activity and spore germination rates were submitted to a chi-squared test.

## Results

### Water Potential of MSR Medium Supplemented With Increasing Concentrations of Polyethylene Glycol

**FIGURE 2:**
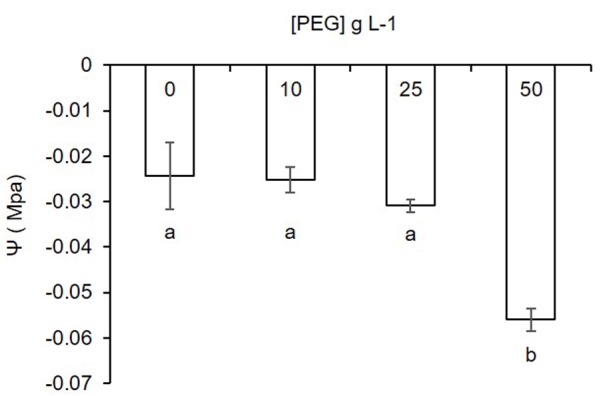
Water potential (Ψ) response of the MSR medium supplemented with increasing concentrations of polyethylene glycol (PEG) (0, 10, 25, and 50 g L^-1^). Data are represented as means ± SD (*n* = 3). Different letters below bars indicate significant differences between treatments using pair-wised comparison (Tuckey’s test, *P <* 0.05).

Water potential of the MSR medium was significantly lower in the PEG^50^ treatment as compared to the Control^-PEG^, PEG^10^, and PEG^25^ treatments (**Figure 2**). No significant differences were noticed between the Control^-PEG^ treatment and the PEG^10^ or PEG^25^ treatments. Modeling of the response by linear regression (data not shown) using a quadratic model showed high statistic fitting (*R*^2^= 0.93501).

### Impact of Decreasing Water Availability on Hyphal Development and Spore Production of *R. irregularis* MUCL 41833

The hyphal length and number of newly produced spores in presence of increasing concentrations of PEG are presented in **Table 1**. After 4 weeks of growth in the HC, the hyphal length was significantly higher in the Control^-PEG^ treatment as compared to the PEG^50^ treatment, but did not differ from the PEG^10^ and PEG^25^ treatments (**Table 1**). No differences were found between the PEG^10^, PEG^25^, and PEG^50^ treatments (**Table 1**).

**Table 1:** Effect of decreasing water availability on (i) hyphal length and spore productions of *R. irregularis* MUCL 41833 developing in the hyphal compartment (HC) of a mycorrhizal donor plant *in vitro* culture system and on (ii) % of germination and germ tube length of isolated spores.

Parameters	Treatments			
	Control^-PEG^	PEG^10^	PEG^25^	PEG^50^
Hyphal length (cm)	1482.6 ± 741.0^a^	1093.7 ± 368.0^ab^	782.8 ± 211.2^ab^	711.1 ± 246.3^b^
Spore number	927.0 ± 180.0^a^	662.7 ± 191.8^ab^	504.8 ± 65.9^b^c	411.5 ± 155.1^c^
Germ tube length (cm)	1.010 ± 0.399^a^	0.584 ± 0.314^b^	0.522 ± 0.289^b^	0.405 ± 0.239^b^
Final germination (%)	93.18^a^	90.69^a^	93.48^a^	20.93^b^

*Treatments consisted in concentrations of 10, 25, and 50 g L^-*1*^ of PEG added in the modified Strullu–Romand (MSR) medium (respectively, PEG^*10*^, PEG^*25*^, and PEG^*50*^) as well as a control without PEG (Control*^-^*^*PEG*^). Hyphal length and number of newly produced spores were estimated 4 weeks after the beginning of the experiment. Percentage of germination and germ tube length of spores were measured at day 110. Data are presented as means ± SD (n* = *6). Different letters indicate significant differences between treatments using pair-wised comparison (Tuckey’s test, P < 0.05).*

The number of newly produced spores was significantly higher in the Control^-PEG^ treatment as compared to the PEG^25^ and PEG^50^ treatments, but did not differ from the PEG^10^ treatment (**Table 1**). Similarly, the number of spores in the PEG^10^ treatment was significantly higher as compared to the PEG^50^ treatment but did not differ from the PEG^25^ treatment (**Table 1**). No significant difference in number of spores was noticed between the PEG^25^ and PEG^50^ treatments (**Table 1**).

### Impact of Decreasing Water Availability on Spore Germination and Germ Tube Length of *R. irregularis* MUCL 41833

The percentage of germinated spores at the end of the experiment (i.e., day 110) was significantly lower in the PEG^50^ treatment (i.e., 20.9%) as compared to the Control^-PEG^ (i.e., 93.2%), PEG^10^ (i.e., 90.7%), and PEG^25^ (i.e., 93.6%) treatments. No significant differences were noticed between the Control^-PEG^, PEG^10^, and PEG^25^ treatments.

**FIGURE 3:**
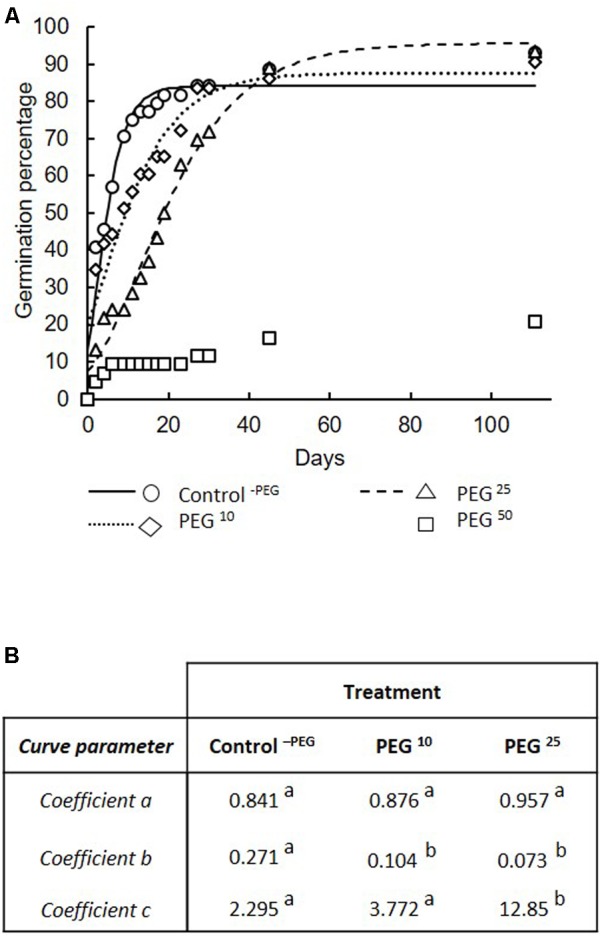
Germination percentage of spores of *R. irregularis* MUCL 41833. **(A)** in response to decreasing water availability. Treatments consisted in concentrations of 10, 25, and 50 g L^-1^ of PEG added to the medium (respectively, PEG^10^, PEG^25^, and PEG^50^) as well as a control without PEG (Control^-PEG^). Germination percentages were modeled using a non-linear regression and a 3-parameter Gompertz model following the equation:y = aexp (–*e*^*-b(x-e)*^). Observed percentages are represented by symbols whereas lines represent modelized curves under Control^-PEG^, PEG^10^, and PEG^25^ treatments. **(B)** Coefficients of the models are estimated in each treatment. Coefficient “a” referred to the cumulated number of germinated spores when the plateau phase was reached, “b” the maximum spore germination rate, and “c” the lag time. Different letters indicate significant differences among treatments using comparison of confidence intervals (see section “Materials and Methods” for further details).

Comparison of modeled germination curves among Control^-PEG^, PEG^10^, and PEG^25^ treatments (**Figure 3A**) demonstrated a significant impact of the treatments (*F* = 29.99, *P <* 0.000001). Parameter “a” (referring to the cumulated number of germinated spores when the plateau phase was reached) did not differ between the control^-PEG^ and the PEG^10^ and PEG^25^ treatments (**Figure 3B**). Parameter “b” (corresponding to the maximum spore germination rate) was significantly higher in the Control^-PEG^ treatment as compared to the two other treatments that did not differ amongst them (**Figure 3B**). Parameter “c” (referring to the lag time) was significantly higher in the PEG^25^ treatment as compared to the Control^-PEG^ and PEG^10^ treatments (**Figure 3B**). No differences were found between Control^-PEG^ and PEG^10^ treatments (**Figure 3B**). The germ tube length of germinated spores under decreasing water availabilities, is presented in **Table 1**. The germ tube length, estimated at 110 days, was significantly lower in the PEG^10^, PEG^25^, and PEG^50^ treatments as compared to the Control^-PEG^ treatment (**Table 1**). No significant differences were found between the three PEG treatments (**Table 1**).

### Impact of Decreasing Water Availability on Pi Uptake by *R. irregularis* MUCL 41833, SDH Activity of Hyphae and Plant Parameters

**FIGURE 4:**
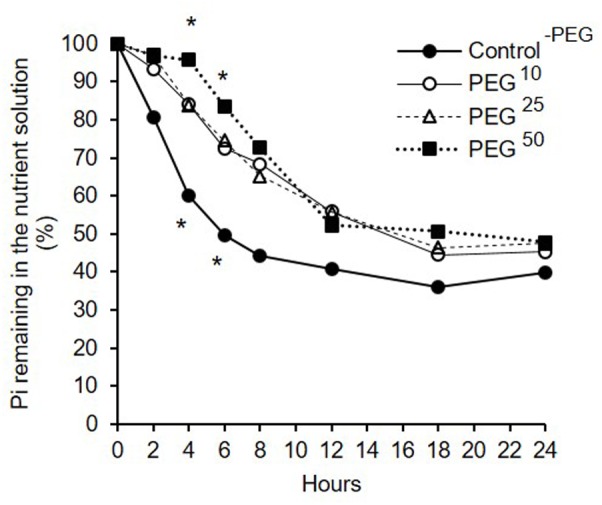
Percentage of inorganic phosphorus (Pi) remaining in the medium in presence of *R. irregularis* MUCL 41833 developing in the HC under decreasing water availabilities during 24 h. Treatments consisted in concentrations of 10, 25, and 50 g L^-1^ of PEG added to the medium (respectively, PEG^10^, PEG^25^, and PEG^50^) as well as a Control^-PEG^. Pi concentrations at each sampling time were standardized after Pi concentrations in the Control^-AMF^ treatment and expressed in percentages relative to their *T*_0_ values (see section “Materials and Methods” for further details). Data are presented as means. At *T*_4_ and *T*_6_, stars above marks represent significant differences among treatments after ANOVA1 analysis followed by pair-wised comparisons (Tuckey’s test, *P <* 0.05, *n* = 6).

Short-term dynamics of Pi uptake (throughout the text expressed as the % of Pi remaining in the medium, see definition in section “Materials and Method”) under decreasing water availabilities is reported in **Figure 4**. Whatever the treatment (Control^-PEG^, PEG^10^, PEG^25^, PEG^50^), the % of Pi remaining in the medium decreased over time suggesting an efficient uptake of Pi by the extraradical hyphae (**Figure 4**).

No significant differences in the % of Pi remaining in the medium at T_24_ was found among treatments. This result is consistent with total SDH activity measured at the end of the experiment (comprised between 72 and 89%) that revealed no significant differences among the treatments (data not shown, *P* = 0.9127).

Analysis of the % of Pi remaining in the medium with repeated measures over the time showed, however, a significant effect of the factor “treatment” (*P <* 0.0001) (**Figure 4**). Further analyses were thus conducted at each time point to compare % of Pi remaining in the medium.

At *T*_4_ and *T*_6_, the % of Pi remaining in the medium in the Control^-PEG^ treatment was significantly lower as compared to the PEG^50^ treatment but did not differ with PEG^10^ and PEG^25^ treatments (**Figure 4**). No significant differences were found between PEG^10^, PEG^25^, and PEG^50^ treatments (**Figure 4**).

Finally, at *T*_2_, *T*_8_, *T*_12_, *T*_18_, and *T*_24_, no significant differences were noticed in the % of Pi remaining in the medium among treatments (respectively *P* = 0.1302, *P* = 0.0853, *P* = 0.5294, *P* = 0.4609, and *P* = 0.4699).

At the end of the experiment, no significant differences were noticed in shoot and roots dry weights (*P* = 0.1557 and *P* = 0.1224, respectively) and water content (*P* = 0.1281 and *P* = 0.119, respectively) (**Table 2**). P concentration in shoots differed significantly among treatments (*P* = 0.0049) (**Table 2**). P concentration in shoots was significantly higher in PEG^50^ as compared to Control^-myco^, Control^-PEG^ and PEG^10^ treatments but did not differ from PEG^25^ treatment (**Table 2**). No differences were found between Control^-myco^, Control^-PEG^, PEG^10^ and PEG^25^ treatments (**Table 2**). No significant differences were found in roots P concentrations (*P* = 0.2944) and in shoots and root P contents (*P* = 0.5672 and *P* = 0.0681, respectively) (**Table 2**).

**Table 2:** Shoot and root dry weights, water content and phosphorus content and concentration [P] of *M. truncatula* plantlets associated to *R. irregularis* MUCL 41833 developing in a HC under decreased water availabilities during 24 h.

		Treatments				
	Plant part	Control^-AMF^	Control^-PEG^	PEG^10^	PEG^25^	PEG^50^
Dry weight (mg)	Shoot	367 ± 61^a^	410 ± 42^a^	342 ± 87^a^	335 ± 39^a^	327 ± 41^a^
	Root	353 ± 43^a^	420 ± 52^a^	358 ± 75^a^	368 ± 49^a^	410 ± 39^a^
Water content (%)	Shoot	74.8 ± 1^a^	73.9 ± 0.9^a^	75.3 ± 1.1^a^	75.7 ± 1.6^a^	76 ± 1.7^a^
	Root	90.2 ± 0.6^a^	90 ± 0.9^a^	89.3 ± 0.8^a^	89.6 ± 1.2^a^	88.8 ± 1.3^a^
P content (mg)	Shoot	0.22 ± 0.05^a^	0.22 ± 0.02^a^	0.21 ± 0.07^a^	0.20 ± 0.05^a^	0.25 ± 0.03^a^
	Root	0.27 ± 0.02^a^	0.29 ± 0.04^a^	0.25 ± 0.03^a^	0.26 ± 0.02^a^	0.27 ± 0.02^a^
[P] (mg Kg^-1^)	Shoot	545 ± 63^b^	522 ± 51^b^	566 ± 104^b^	571 ± 143^ab^	714 ± 49^a^
	Root	749 ± 76^a^	671 ± 112^a^	679 ± 113^a^	663 ± 65^a^	642 ± 48^a^

*Treatments consisted in concentrations of 10, 25, and 50 g L^-*1*^ of PEG added to the medium (respectively, PEG^*10*^, PEG^*25*^, and PEG^*50*^) as well as a control without PEG (Control*^-^*^*PEG*^). Data are presented as means ± SD (n* = *6). Different letters indicate significant differences between treatments using pair-wised comparison (Tuckey’s test, P < 0.05).*

## Discussion

Drought stress is an increasing problem in agriculture and methods to alleviate or at least decrease its impact on crops are highly demanded. Plant roots are intimately associated with microorganisms. Some of them (e.g., AMF) have been reported to increase the resistance/tolerance of plants to environmental stresses such as water deficit (Jayne and Quigley, 2014; Fernández-Lizarazo and Moreno-Fonseca, 2016). A few studies conducted within field also reported significant increase of plant biomass under dry environments (Caravaca et al., 2003; Querejeta et al., 2006; Pellegrino et al., 2011). This suggested that the utilization of AMF may potentially represent an innovative approach to address the issue of decreasing crop production under drought stress conditions. If the beneficial effects of AMF on plants under water stress have been reported (Jayne and Quigley, 2014; Fernández-Lizarazo and Moreno-Fonseca, 2016), limited knowledge is available on the impact of decreasing water availability on the development of AMF and their capacity to take up and transport Pi to plants. Here we reported on the impact of decreasing water availability on the development of *R. irregularis* MUCL 41833 and its capacity to take up and transport Pi under *in vitro* culture conditions. Water deficit was established via the application of increasing amounts (0, 10, 25, and 50 mg L^-1^) of PEG to the medium (corresponding to a decrease in water potential in a range from -0.024 to -0.056 Mpa). At the highest concentration of PEG and thus lowest availability of water, hyphal development, and spore production, as well as spore germination were decreased. Similarly, the dynamic of Pi depletion in the root-free HC was impacted at the highest concentration of PEG, even if total depletion was similar at the end of the experiment (i.e., after 24 h) suggesting an effective but slower dynamics of Pi uptake by the ERM.

### Establishment of Water Availability Conditions to Study the Impact of PEG on *R. irregularis* MUCL 41833

Water potential measured in the MSR medium decreased inversely to PEG concentration and accordingly to previous results followed a quadratic model (Michel and Kaufmann, 1973). PEG significantly decreased the water potential of the MSR medium at the concentration of 50 g L^-1^. Conversely, 10 and 25 g L^-1^ did not significantly decrease the water potential as compared to the Control^-PEG^ treatment. Water potentials considered in our experiment (from -0.024 to -0.056 Mpa) were above the limit of what is considered a drought stress environment (<-1 Mpa). However, it was sufficient to establish a gradient of water availability to the fungus impacting at the highest concentration development and to a lesser extend Pi uptake by the AMF. Higher concentrations of PEG in the MSR medium were also tested and decreased further the water potential (see **Supplementary Figure S1**). However, under these conditions, the MSR medium did not solidify completely, probably due to a decreased polymerization of the gelling agent (i.e., Gelrite) as earlier reported by van der Weele et al. (2000). An increased opacity of the medium was also noticed, preventing the easy and rigorous observation of the AMF.

### Impact of Decreasing Water Availability on Fungal ERM Development

Hyphal length was significantly decreased in the PEG^50^ treatment and spore production in the PEG^25^ and PEG^50^ treatment as compared to the Control^-PEG^ treatment. These results were consistent with the observations of Khalvati et al. (2005) in a pot experiment conducted in the greenhouse. These authors noticed that the extraradical hyphae density of *G. intraradices* was decreased under drought conditions. Conversely, Bethlenfalvay et al. (1988) and Davies et al. (1992) noticed an increase in hyphal length of *Glomus mosseae* and *Glomus deserticola*, respectively under greenhouse pot experiments. Similar contrasting results were reported for spores production. Drought drastically decreased their number in *Gigaspora albida* and *Scutellospora heterogama* (Silva et al., 2015), *Entrophospora colombiana* and *Acaulospora longula*, (Sieverding and Toro, 1988), while no effects were noticed on *Gigaspora margarita, Glomus clarum*, and *G. mosseae* (Sylvia and Schenck, 1983) under greenhouse pot experiments. Spore germination was also significantly decreased in the PEG^50^ treatment as compared to the other treatments and germ tube length was significantly decreased at all PEG concentrations as compared to the Control^-PEG^ treatment. This corroborates the results of Sylvia and Schenck (1983) and Douds and Schenck (1991). These authors, respectively, reported a decrease in germination of *G. intraradices, G. mosseae*, and *A. longula* spores placed in soil substrate of water potentials equal or lower than -0.5 MPa and a decrease in germination of *G. clarum, G. etunicatum*, and *G. macrocarpum* spores placed in soil substrate of water potentials below -0.01 Mpa. Under *in vitro* conditions, Estaun (1989) observed a negative effect of glycerol-induced osmotic stress on germination and germ tube length of *G. mosseae* starting at a water potential of -0.21 Mpa.

Noticeably, these results clearly demonstrated that the impact of decreasing water availability can drastically differ between AMF species. Although water potentials in the above-mentioned studies were much lower than in our experiment, *R. irregularis* MUCL 41833 seemed to be very sensitive to water availability as noticed by the decreasing hyphal growth, spore production and spore germination obtained in the PEG^50^ treatment (corresponding to -0.056 Mpa). In previous greenhouse pot experiments the effects of drought on the decrease in spore production and extraradical development could not be strictly attributed to a direct impact on the fungus. Indeed, it was not possible to separate fungus from plant and thus direct from indirect effect on the fungus. Nevertheless, the authors suggested that the effects were due to a decrease of the carbon sink constituted by the plants (Augé, 2001; Manoharan et al., 2010; Silva et al., 2015). In our study conducted strictly *in vitro*, thus avoiding any confounding effects, only the ERM of the fungus was exposed to a decrease in water availability. The results clearly demonstrated that *R. irregularis* was directly impacted by a decrease in water availability. The effects observed were thus only attributable to the water potential and the resulting osmotic stress.

### Impact of Decreasing Water Availability on Pi Uptake and Transport by the AMF

One of the most noticeable attribute of AMF is the ability of the fungus to take up and transport Pi to its host. The *in vitro* culture system used in this study was particularly adequate to investigate Pi uptake by the AMF as earlier demonstrated (Nielsen et al., 2002; Zocco et al., 2011; Calonne et al., 2014) but was never applied under conditions of decreased water availability. Under PEG-induced decrease in water availability, Pi uptake by the fungus was significantly impacted at the lowest water availability (PEG^50^), while no impact was noticed for PEG^10^ and PEG^25^. Indeed, within the first 6 h, the % of Pi remaining in the medium was lower in the HC of the Control^-PEG^ treatment as compared to the PEG^50^ treatment indicating a probable effect of lower water availability on the Pi uptake and possibly on the Pi translocation to the plant. Interestingly, at the end of the experiment, no differences were noticed in % of Pi remaining in the medium whatever the water availability. Since SDH activity of the mycelium was similar between the different treatments, the decrease in Pi uptake by the fungus within the first hours cannot be clearly attributed to the death of a substantial proportion of hyphae. We hypothesized that the decreased rate of Pi uptake by the fungus could be related with a lower diffusion of Pi due to the viscosity of PEG solution or to an impact on the Pi transporters in the hyphae. It was, indeed, reported that under lower phosphate concentrations in the environment, the expression of the fungal Pi transporter *GintPT* was decreased (Maldonado-Mendoza et al., 2001). PEG could decrease water mass-flow and Pi diffusion to the hyphae causing *GintPT* to decrease its expression. The lower Pi uptake may be accompanied by a slower translocation within the hyphae, although this needs to be demonstrated.

### Effect of Decreasing Water Availability on Plant Parameters of *M. truncatula*

No significant difference in shoots and roots P content was found in the absence (Control^-AMF^) or in the presence (Control^-PEG^, PEG^10^, PEG^25^, and PEG^50^ treatment) of mycelium in the HC. These results were expected as no differences in the Pi uptake by the mycelium could be detected among treatments after 24 h. Furthermore, it is unlikely that Pi uptake by the hyphae could directly impact P content in plants. Indeed, Pi quantities taken up by the hyphae are very small compared to the whole plant P content. Moreover, the short time of experiment might not be sufficient to observe a complete translocation of Pi to the plant. Indeed, it has been demonstrated that Pi accumulated in symbiotic structures in the RC only after 37 h (Nielsen et al., 2002).

Surprisingly, we found that P concentrations in shoots were significantly increased under PEG^50^ treatment compared to the Control^-AMF^ (×1.32), Control^-PEG^ (×1.37), and PEG^10^ (×1.26), treatments. Shoot P content was expected to follow the same trend given that shoot biomass was not found significantly different among treatments. However, shoot P content did not differ significantly among treatment, probably because of the impact of high standard deviations. As no difference was found in root P concentration among treatments, increased P concentration in shoots under the PEG^50^ treatment might be due to a higher root to shoot P translocation compared to the other treatments. In a similar experiment (Gil-Cardeza et al., 2017), an increased translocation of P from root to the shoot was also observed. Pi uptake by the mycelium was monitored in response to formaldehyde applied in the HC. After 24 h, P concentration in shoots of *M. truncatula* was significantly increased (×1.9) as compared to the treatment without stress. This effect was explained by a plant response to the high stress applied on the mycelium in the HC. Although the role of AM symbiosis in the plant P translocation is still barely known, some studies point the interaction of AM symbiosis long distance signals with the plant P homeostasis regulation. Analysis of miRNA expression pattern in mycorrhized *M. truncatula* plants under low phosphate conditions showed that AMF could increase the expression of miR399 (Branscheid et al., 2010), a P starvation signal involved in the regulation of root-shoot P compartmentation in the *Arabidopsis thaliana* model (Doerner et al., 2008). Moreover, AM symbiosis can up or down regulate the expression of numerous Pht1-type P transporter in plants and are believed to play a possible role in P translocation (Javot et al., 2007).

It was demonstrated that extraradical hyphae could directly transfer soil water to the plant and improve plant water status (Khalvati et al., 2005). However, the presence of ERM in the HC seemed not to improve water status of the plant as no significant increase in shoots and roots water content of *M. truncatula* were found. Similarly, no effect was found by adding PEG while Li et al. (2013) demonstrated that PEG addition in the HC enhanced expression of the fungal aquaporin genes *GintAQPF1* and *GintAQPF2* of *R. irregularis* and may increase water absorption by the hyphae. The absence of effect of the treatments on water content, in shoots and roots of *M. truncatula* in our experiment was expected because of the relative short time application of stress (24 h). Another explanation is that PEG concentrations tested were not as severe as water potential level found under a drought environment.

## Conclusion

The present study cannot be compared *per se* with drought conditions in natural environments as the water potentials tested in the *in vitro* system were not low enough to induce drought stress in soil. However, the *in vitro* culture system was adequate to investigate the impact of a gradient of water availability on various fungal parameters and function (i.e., Pi uptake and transport) under strict controlled conditions. PEG-induced decrease in water availability impacted hyphal development and spore production, spore germination and Pi uptake dynamics at the highest concentration suggesting a probable higher effect under even more limiting water conditions. The *in vitro* system used appeared adequate to investigate various parameters of the AMF. It opens the door for comparative studies including AMF strains isolated from drought environments as well as for molecular studies involving expression of aquaporin genes or genes involved in stress tolerance.

## Author Contributions

OLP contributed to the development of experiments, data collection, analysis, and interpretation of the data, drafted the work, and gave the final approval and agreed with all aspects of the work. SD made substantial contributions to the conception and design of the experiments, interpretation of the data, draft corrections, and gave the final approval and agreed with all aspects of the work.

## Conflict of Interest Statement

The authors declare that the research was conducted in the absence of any commercial or financial relationships that could be construed as a potential conflict of interest.

## Footnotes

1 www.mycorrhiza.be/ginco-bel

## Supplementary Material

FIGURE S1 Water potential (Ψ) response of the Modified Strullu–Romand (MSR) medium supplemented with sugar (3 g L^-1^) and increasing concentrations of Polyethylene glycol 8000 (0, 50, 75, 130, and 200 g L^-1^). Data are represented as means ± SD (*n* = 3).
