# Supplementary figures and images for: Reducing Water Availability Impacts the Development of the Arbuscular Mycorrhizal Fungus Rhizophagus irregularis MUCL 41833 and Its Ability to Take Up and Transport Phosphorus Under in Vitro Conditions
Source: Front Microbiol. 2018 Jun 11;9:1254. doi: 10.3389/fmicb.2018.01254 (PMC6004939; doi:10.3389/fmicb.2018.01254)

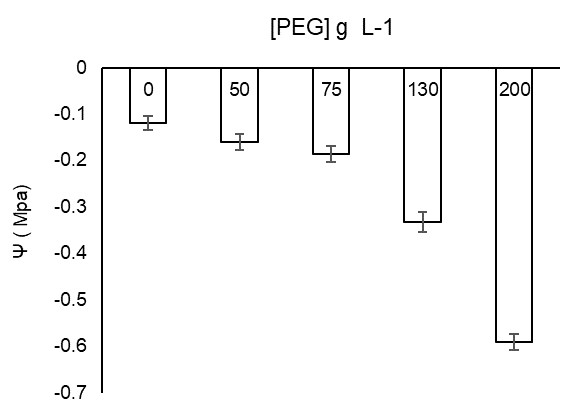

Supplement: FIGURE S1 — Water potential (Ψ) response of the Modified Strullu–Romand (MSR) medium supplemented with sugar (3 g L-1) and increasing concentrations of Polyethylene glycol 8000 (0, 50, 75, 130, and 200 g L-1). Data are represented as means ± SD (n = 3). [file Image_1.JPEG]
